# Biological roles of Yin Yang 2: Its implications in physiological and pathological events

**DOI:** 10.1111/jcmm.15919

**Published:** 2020-09-23

**Authors:** Lang Li, Yanjun Li, Ian Timothy Sembiring Meliala, Vivi Kasim, Shourong Wu

**Affiliations:** ^1^ Key Laboratory of Biorheological Science and Technology Ministry of Education College of Bioengineering Chongqing University Chongqing China; ^2^ The 111 Project Laboratory of Biomechanics and Tissue Repair College of Bioengineering Chongqing University Chongqing China; ^3^ State and Local Joint Engineering Laboratory for Vascular Implants Chongqing China

**Keywords:** cancer, development, immune response, YY family, YY2

## Abstract

Yin yang 2 (YY2) is a multifunctional zinc finger protein that belongs to the yin yang (YY) family. YY2 has dual function in regulating gene expression, as it could act either as a transcriptional activator or as a repressor of its target genes. YY2 could regulate genes that have been previously identified as targets of yin yang 1 (YY1), another member of the YY family, by binding to their common binding sequences. However, recent studies revealed that YY2 also has its own specific binding sequences, leading to its particular biological functions distinct from those of YY1. Furthermore, they have different levels or even opposite regulatory effects on common target genes, suggesting the importance of balanced YY1 and YY2 regulations in maintaining proper cellular homeostasis and biological functions. Recent studies revealed that YY2 plays crucial roles in maintaining stemness and regulating differentiation potential of embryonic stem cells, as well as in the development of the brain, nervous and cardiovascular systems. YY2 expression is also closely related to diseases, as it could act as a tumour suppressor gene that regulates tumour cell proliferation and metastasis. Moreover, YY2 is also involved in immune regulation and immune surveillance. Herein, we summarize recent perspectives regarding the regulatory functions of YY2, as well as its biological functions and relation with diseases.

## 1 INTRODUCTION

**FIGURE 1:**
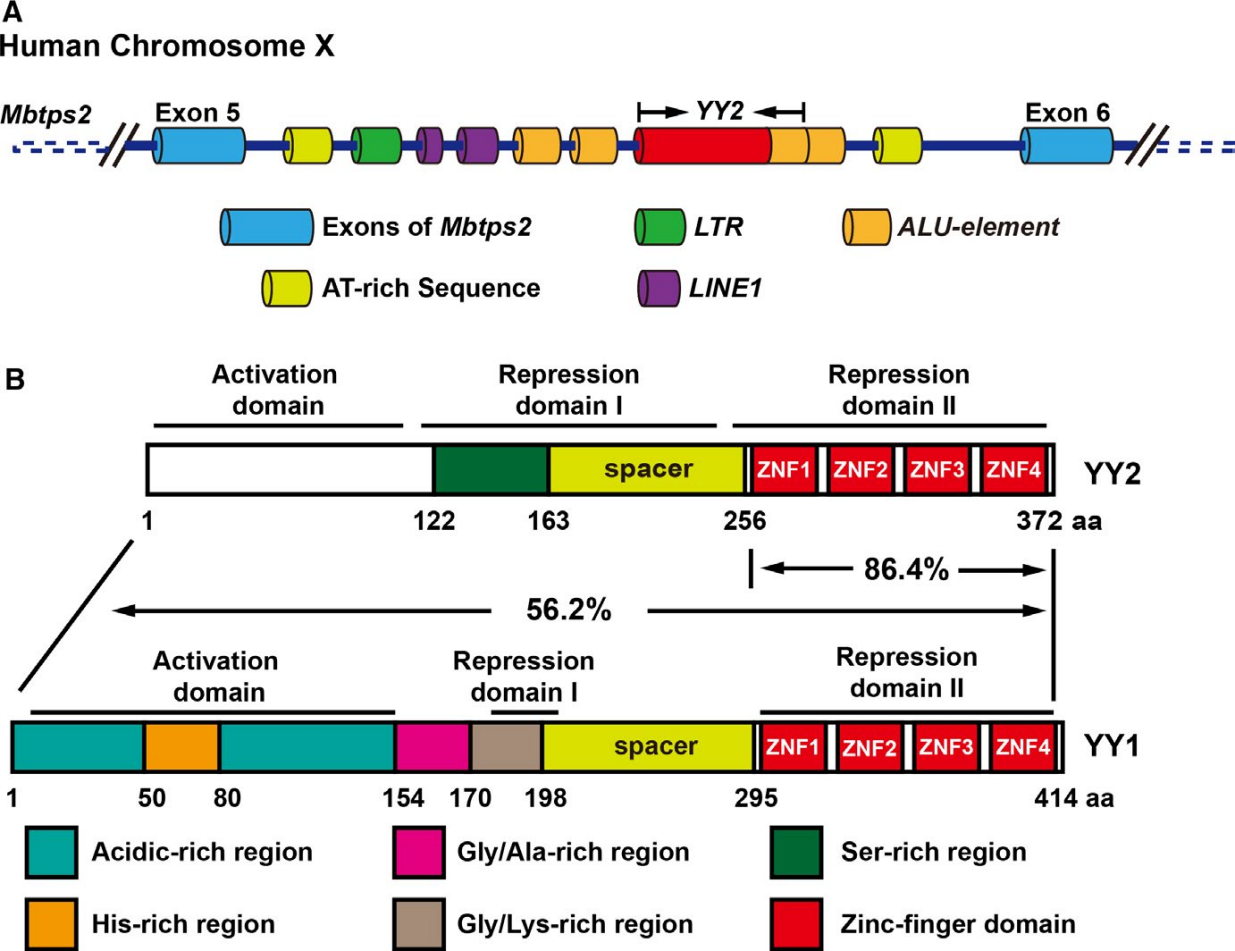
Schematic diagram of YY2 gene and YY2 protein. A, Location of YY2 gene. The region of YY2 gene and its surrounding region on human chromosome X is shown. LTR, long terminal repeat; ALU, Alu‐element; LINE1, long interspersed nuclear element 1. B, Comparison between the protein structures of YY2 and YY1. The percentages of identity between the entire proteins and between the zinc fingers of the two proteins are indicated. Ser, serine‐rich domain; His, histidine‐rich domain; acidic: acidic‐rich domain; GA, glycine/alanine‐rich domain; GK, glycine/lysine‐rich domain

*Yin yang 2* (*YY2*) is a retrotransposon‐derived paralogue of *yin yang 1* (*YY1*).^1^ *YY2* originated from YY1 mRNA, which was retrotransposed and inserted into the intronic segment between exons 5 and 6 of *membrane‐bound transcription factor peptidase site 2* (*Mbtps2*) gene, surrounded by genomic elements such as long terminal repeat (LTR), Alu‐element (ALU), long interspersed nuclear element 1 (LINE1) and AT‐rich domains (Figure 1A). This retrotransposition occurred after the divergence of placental mammals from other vertebrates.^1^ Thus, unlike *YY1,* which is conserved in all vertebrate lineages,^1^ *YY2* is conserved only in placental mammals.^1^, ^2^ Together with *YY1* and *reduced expression 1* (*REX1*), YY2 belongs to yin yang (YY) family.^3^, ^4^, ^5^ *YY2* encodes a protein with 378 amino acid that belongs to GLI‐Krüppel class protein with four C2H2 zinc finger domains, sharing 56.2% overall identity with YY1.^3^ The conservation is even higher in their zinc finger domain regions, as YY2 zinc finger domain shares almost 86.4% identity with that of YY1 (Figure 1B).^3^

**FIGURE 2:**
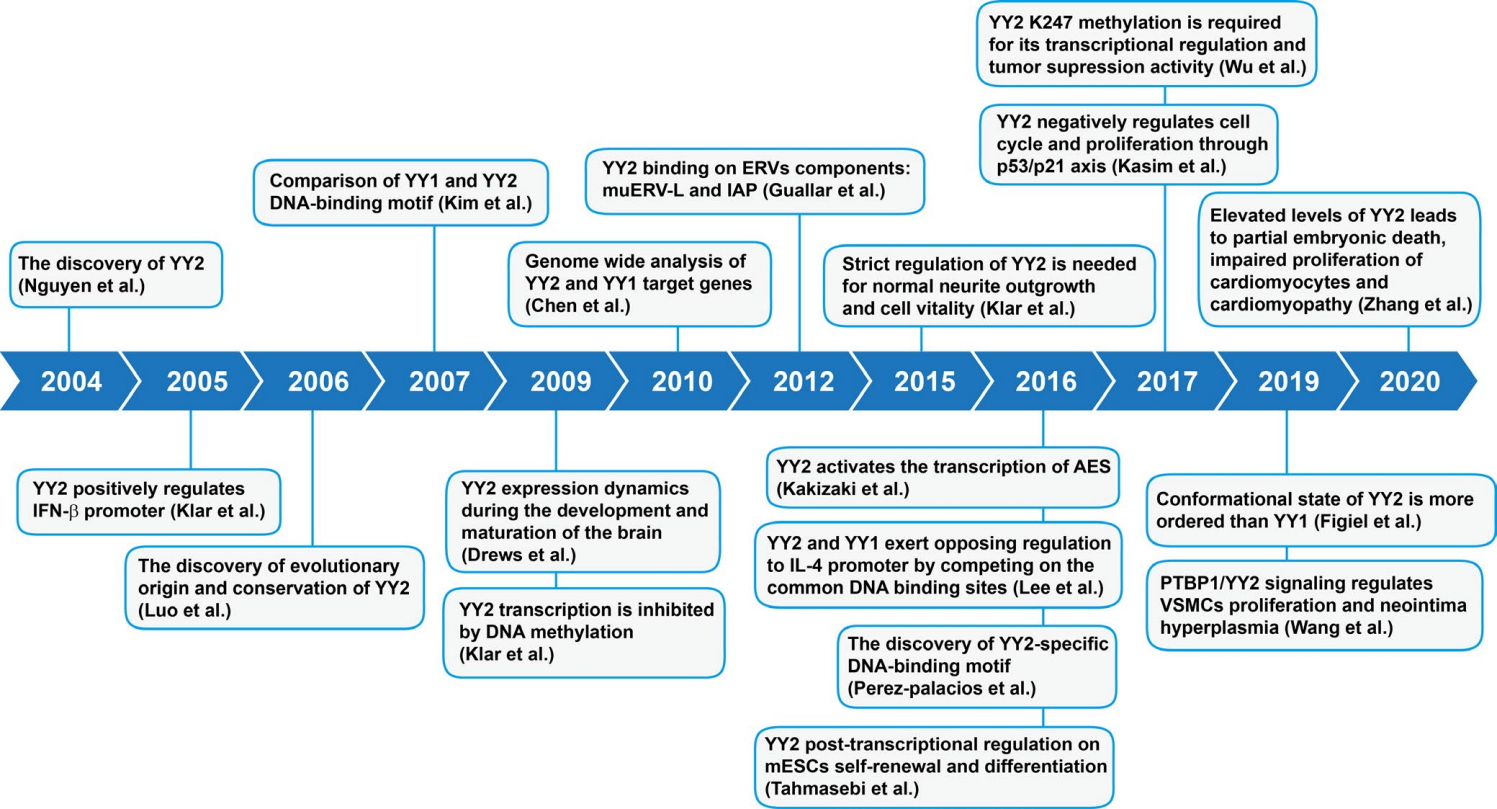
Timeline of major discoveries regarding the regulatory and the biological functions of YY2. Outline describing major discoveries of the regulation of YY2 and the physiopathological roles of YY2 since its discovery in 2004 is shown

Since its discovery by Nguyen *et al* in 2004, studies have revealed the complex regulatory mechanisms of YY2 expression as well as its mechanisms in regulating target genes; furthermore, increasing evidences showed that YY2 is crucial for various physiological and pathological pathways (Figure 2). By deletion analysis, Nguyen *et al* found that the N‐terminus (32‐102 residues) of YY2 might be a potential transcriptional activation region, while C‐terminus (237‐372 residues) might be a potential transcriptional inhibition region.^3^ These findings suggest that similar to YY1, YY2 might also have both transcriptional activation and repression activities. Indeed, previous studies provide evidences regarding these regulatory activities, as YY2 could activate the transcriptional activity of *amino‐terminal enhancer of split* (*AES*) and tumour suppressors *p53*,^6^, ^7^ while suppressing that of *interleukin‐4* (*IL‐4*)^8^ and several pluripotency factors including *organic cation/carnitine transporter‐4* (*Oct4)*, *oestrogen‐related receptor beta* (*Esrrb)*, *tet methylcytosine dioxygenase 1* (*Tet1*) and *tet methylcytosine dioxygenase 2* (*Tet2*).^9^ On the other hand, a recent report demonstrated that the N‐terminus of YY2 has a more ordered characteristic compared to YY1, implying the possibility of the different regulatory functions of the two proteins.^10^

Previous studies revealed that YY1 is ubiquitously expressed and could regulate more than 7% of all vertebrate genes,^11^, ^12^ and thus plays crucial roles in various biological and physiological functions. Furthermore, aberrant YY1 expression is closely related to various diseases.^7^, ^13^, ^14^, ^15^, ^16^, ^17^, ^18^ Similar to the YY1 protein, as shown by Drews *et al*, the expression of YY2 might be ubiquitous as well since YY2 mRNA could be detected in the entire embryonic mice.^19^ Indeed, YY2 is expressed in various types of tissues, including the cardiovascular, neuronal, breasts, muscle, eye, stomach, lung, brain and testis.^1^, ^7^, ^9^, ^19^, ^20^

More recent studies have revealed the unique functions of YY2 in embryogenesis and in the development of brain, nervous and cardiovascular systems, as well as in the immune system regulation.^8^, ^9^, ^21^, ^22^ Furthermore, we and other groups have demonstrated that YY2 is crucial in regulating tumour generation and progression, as it is critical in regulating tumour cell proliferation, cell cycle arrest, metastasis, and metabolic reprogramming. Interestingly, the roles of YY2 in tumorigenesis are antagonistic to those of YY1, suggesting that YY2 might be a potential tumour suppressor.^6^, ^7^, ^23^ These facts suggest the importance and specific physiopathological functions of YY2. In this review, we will highlight the regulatory functions of YY2, as well as its biological and physiological functions and potential roles in regulating disease progression.

### 1.1 YY2 is a multifunctional regulator

#### 1.1.1 Multi‐stages regulation of YY2 expression

While recent studies have revealed several mechanisms regarding the molecular regulatory mechanism of YY2 expression, many details are still unknown. Changes in the YY2 expression level could be observed during development and pathological conditions. For instance, YY2 expression level shows a dynamic fluctuation during brain development and significantly decreases during tumour progression.^6^, ^7^, ^19^ Despite its dynamic changes in physiological and pathological conditions, the mechanism regulating its transcriptional activity remains unelucidated.

Another intriguing, unravelled matter regarding *YY2* transcriptional regulation is whether it is regulated simultaneously with that of *Mbtps2*. As mentioned above, *YY2* originated from YY1 mRNA which was retroposed into *Mbtps2* gene.^1^, ^2^ Previous studies suggested two contradictive possibilities that need further clarification: some evidences support the hypothesis that *YY2* and *Mbtps2* are regulated simultaneously, while others support the hypothesis that they are regulated separately. *YY2* and *Mbtps2* showed similar spatial expression patterns in the brain, ovary and testis, as well as in breast cancer cell lines, indicating that these two genes might be subjected to similar transcriptional control.^1^, ^7^ However, other studies demonstrated that while YY2 expression shows dynamic changes in neocortex and cerebellum during development, Mbtps2 remains unchanged.^19^ Furthermore, as will be described below, while epigenetic regulation is an important mechanism in regulating YY2 expression, the expression of *Mbtps2* could not be regulated by DNA methylation.^24^ These contradictive results make the molecular mechanism of *YY2* transcriptional regulation more complex.

**FIGURE 3:**
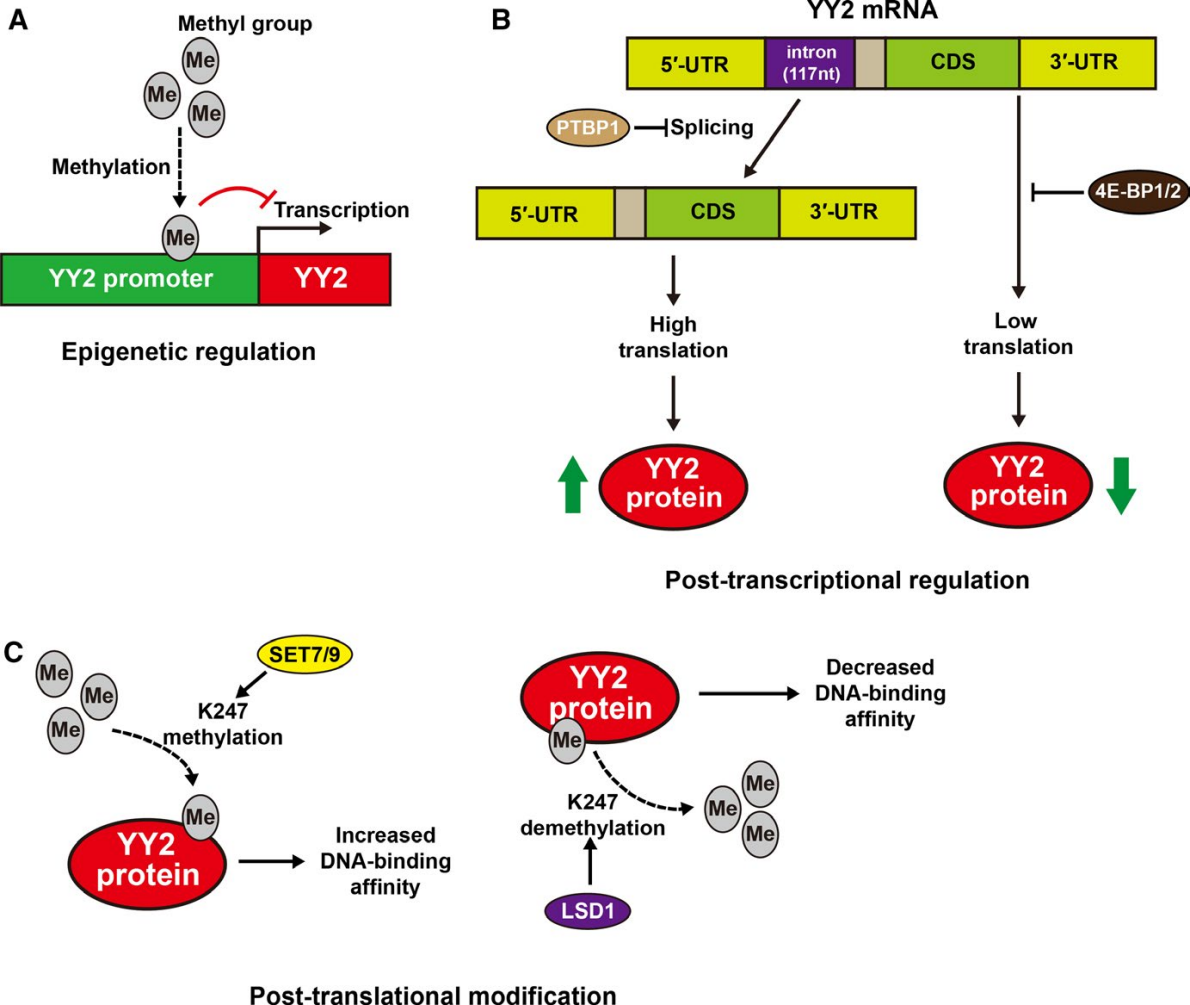
Multi‐stages regulation of YY2 expression. A, Epigenetic regulation of YY2 gene. Human YY2 gene transcription is inhibited by DNA methylation. B, Post‐transcriptional regulation of YY2 mRNA. Posttranscriptional regulation of YY2 expression occurs by two steps: (i) PTBP1 suppresses the splicing of the YY2 5’‐UTR, leading to the generation of YY2 splicing variant with higher intron retention; and (ii) 4E‐BP1/2 suppresses the translation of the YY2 mRNA with the suppression effect depends on the intron retention degree. C, Post‐translational modification of YY2 protein. YY2 protein is methylated by SET7/9 and demethylated by LSD1. Methylation of YY2 increases its binding affinity to the promoters of its target genes

Studies showed that epigenetic regulation is critical for *YY2* expression. Klar *et al* found that the expression of human *YY2* is controlled by its adjacent 5’‐promoter region. They found that the proximal *YY2* promoter is hypermethylated to maintain its low expression, while treatment with 5‐Aza‐2‐deoxycytidine, a reagent that induces DNA demethylation by constitutively inhibiting DNA‐methyltransferases, significantly increases it, suggesting that YY2 expression could be controlled by epigenetic regulation through its promoter methylation (Figure 3A).^24^

Besides epigenetics and transcriptional regulations, post‐transcriptional regulation is an important regulatory pathway of YY2 expression. Mouse embryonic stem cells (mESCs), in which the level of YY2 is strictly regulated, provide a good example of this type of regulation. A recent study by Tahmasebi *et al* demonstrated that YY2 is essential for mESC self‐renewal, and for directing the differentiation of mESCs towards cardiovascular lineages.^9^ As mESCs differentiate towards mouse embryoid bodies (EBs), the degree of intron retention of YY2 5′‐UTR decreases, while the alternative splicing of YY2 increases. Additionally, they found that the splicing regulator polypyrimidine tract‐binding protein 1 (PTBP1) dramatically suppresses YY2 5′‐UTR splicing, resulted in the addition of 117 nucleotides. The retained region possesses a GU dinucleotide at the 5′ splice site and an AG dinucleotide at the 3′ splice site, thus maintaining the characteristics of an intron and increases the complexity of YY2 mRNA secondary structure. This, in turn, leads to an increased sensitivity to *4E‐binding proteins* (4E‐BP)‐mediated translational repression and limits YY2 expression at a low level in mESCs, which is critical for maintaining its stemness (Figure 3B).^9^ Furthermore, comparison analysis between YY2 expression levels in different tissues also showed that the highest expression of YY2 protein could be detected in the heart and muscle tissues, where the YY2 5′‐UTR intron retention is at the lowest levels, indicating that such negative regulation of YY2 protein level could be observed across different tissues.^9^ Together, these facts suggest that precise control of YY2 expression level could be achieved by alternative splicing regulation and is crucial in determining cell fate, *that is*, maintenance of stemness and differentiation towards a more mature tissue.

Post‐translational modification is also a crucial regulation for YY2 protein. Indeed, as shown in Figure 3C, methylation modification of YY2 protein is critical for its DNA‐binding activity and tumour‐suppressive effect.^23^ Together, YY2 expression is regulated by a complex, multi‐stage mechanism closely related to its biological and pathological functions, and further investigation is needed to figure out their details.

#### 1.1.2 YY2‐mediated transcriptional regulation

As shown in Table 1, YY2 is a multifunctional transcription regulator that can both activate and inhibit the transcription of various target genes. Due to the high structural homology in their zinc finger regions, YY2 and YY1 share a common DNA‐binding site 5′‐CGCCATnTT‐3′ (core binding site: 5′‐CCAT‐3′).^2^ Consequently, YY2 could regulate several genes which are originally found to be regulated by YY1, such as *Myc proto‐oncogene* (*c‐Myc*), *Fos proto‐oncogene* (*c‐Fos*) and *C‐X‐C motif chemokine receptor 4* (*CXCR4*), by interacting with their promoters through DNA‐binding site common with that of YY1.^3^ Interestingly, YY2 could exert functions antagonistic to that of YY1 in regulating their common target genes. For instance, YY2 alleviates the suppressive effect of YY1 on *beta interferon* (*IFN‐β*) promoter activity,^21^ as well as the enhancing effect of YY1 on *IL‐4* promoter activity.^8^ In some cases, for example, in the regulation of *c‐Myc* and *CXCR4*, YY2 exerts either agonistic or antagonistic function to YY1, as YY2 activates their transcriptional activities at low dose and suppresses them at high dose; while in the regulation of *c‐Fos*, YY2 regulatory effect is more prominent at low dose,^3^ suggesting a dose‐dependent regulation of YY2 on its target genes.

**TABLE 1:** Human (mouse) genes regulated by YY2

Genes	Regulation	Species	Cell types	Functions	References
*AES*	Activation	Human	HCT116, RKO	Suppresses tumour metastasis	^6^
*BCL2L11*	Repression	Human	HeLa	Pro‐apoptosis	^40^
*CD36*	Repression	Human	HeLa	Tumour metabolism	^40^
*CDC2*	Repression	Human	HeLa	Cell cycle regulator	^40^
*Cdkl2*	Not determined^*^	Mouse	B7	Epithelial‐Mesenchymal Transition	^25^
*c‐Fos*	Activation^**^	Human	HeLa	Cell proliferation, differentiation, and transformation	^3^
*c‐Myc*	Activation (L)^***^ Repression (H)	Human	HeLa	Cell cycle regulator, apoptosis and transformation	^3^
*CXCR4*	Activation (L)^***^ Repression (H)	Human	HeLa	Tumour angiogenesis	^3^
*Esrrb*	Repression	Mouse	ESCs	Stemness, pluripotency	^9^
*Fras1*	Not determined^*^	Mouse	B7	Tumour metastasis	^25^
*Gcnt2*	Not determined^*^	Mouse	B7	Epithelial‐Mesenchymal Transition	^25^
*IAP*	Activation	Mouse	ESCs, TCs	Differentiation and morphogenesis	^25^
*IFN‐β*	Competition with YY1	Mouse	LM^Tk^	Immunosurveillance	^25^
*IL‐4*	Activation	Mouse Human	EL‐4 T HEK293	Immune & inflammatory responses	^8^
*MLC2a*	Activation	Mouse	Embryoid bodies	Cardiovascular development	^9^
*MLC2v*	Activation	Mouse	Embryoid bodies	Cardiovascular development	^9^
*muERV‐L*	Activation	Mouse	ESCs, TCs	Differentiation and morphogenesis	^23^
*MYPN*	Repression	Human	HeLa	Cardiovascular development	^23^
*NKx2*.*5*	Activation	Mouse	Embryoid bodies	Cardiovascular development	^9^
*Oct4*	Repression	Mouse	ESCs	Stemness, pluripotency	^9^
*OLR1*	Repression	Human	HeLa	Tumour metastasis	^23^
*p21*	Activation	Human	HCT116, MCF‐7, HepG2	Cell cycle regulation	^7^
*p53*	Activation	Human	HeLa, HCT116, MCF‐7, HeLa	Cell cycle regulation, DNA damage repair, apoptosis, tumour metabolic reprogramming	3, 7
*PDE2A*	Repression	Human	HeLa	Tumour metastasis	^23^
*PTGS2*	Repression	Human	HeLa	Tumour metastasis	^23^
*Tet1*	Repression	Mouse	ESCs	Stemness, pluripotency	^9^
*Tet1*	Repression	Mouse	ESCs	Stemness, pluripotency	^9^
*Tet2*	Repression	Mouse	ESCs	Stemness, pluripotency	^9^
*αMHC*	Activation	Mouse	Embryoid bodies	Cardiovascular development	^9^

* The reference only showed that YY2 binds to the promoter of the indicated gene using chromatin immunoprecipitation assay.

** YY2 activity in regulating the indicated gene is more prominent at low dose.

*** YY2 activates the transcription of the indicated gene at low dose, and suppresses it at high dose. L, low dose of YY2; H, high dose of YY2.

An earlier study showed that the slight difference in the zinc finger regions of YY2 and YY1 results in weaker YY2 DNA‐binding affinity compared to that of YY1 on their common DNA‐binding site. However, a more recent study showed that YY2 DNA‐binding affinity on their common DNA‐binding site in the *AES* promoter is stronger than YY1. In addition, YY2 could directly activate the transcription of *AES*, while YY1 requires the presence of a co‐activator to exert its transcriptional activity.^6^ The mechanism underlying this, as well as whether a stronger YY2 DNA‐binding affinity compared to YY1 could be found in other target genes or is specific to *AES*, remains unknown. Nevertheless, while its detailed mechanism needs to be elucidated further, these findings confirm that YY2 and YY1 have different binding affinity to their common target genes.

Despite sharing common DNA‐binding site and target genes with YY1, YY2 also has its own, specific target genes, as sequential differences in zinc finger region between YY2 and YY1 enables YY2 to bind to its specific DNA‐binding site (5′‐AnAGAAGTGG‐3′).^2^, ^25^ Our previous study demonstrated that YY2 binds to *p53* promoter region through its specific DNA‐binding site to which YY1 could not bind, leading to the activation of p53 transcription and increased protein expression.^7^ In contrast, YY1 regulation on p53 occurs by facilitating the binding between p53 protein and its negative regulator mouse double‐minute 2 (MDM2), leading to ubiquitination‐mediated proteasomal degradation, and subsequently, the decrease of p53 protein accumulation.^26^ Furthermore, through its specific DNA‐binding site, YY2 could also bind to the promoters of *cyclin‐dependent kinase‐like 2* (*Cdkl2*), *fraser extracellular matrix complex subunit 1* (*Fras1*) and *glucosaminyl (N‐acetyl) transferase 2* (*Gcnt2*); however, the regulatory effects and the phenotypes induced by these regulations remain to be elucidated.^25^

YY2 transcriptional regulatory function is not only associated with the expression of the coding genes, but also linked to the expression of long noncoding RNA (lncRNA) genes. For instance, the expression levels of lncRNA C230088H06‐Rik‐202 and Gm26624 are positively regulated by YY2 through its specific binding site on their promoters.^25^ Although the detailed mechanism of YY2 regulation on these genes has not been fully elucidated, the identification of YY2‐specific DNA‐binding site in vivo suggests a broad scope of YY2 target genes.^2^, ^25^

The transcriptional activity of YY2 is inseparable from its protein structure. Although YY2 is structurally highly homologous to YY1, it still has a 43.8% non‐homologous region, which is dominantly located at the N‐terminus of its protein.^3^ Furthermore, unlike YY1, YY2 does not contain the acidic‐rich domain that maximizes its transcriptional activation capacity. A recent report also showed that compared to YY1, the N‐terminus of YY2 has a more stable structure. This less disordered characteristic of N‐terminus might underlie the capability of YY2 to interact with a more limited range of cofactors compared to YY1.^10^ While further investigation is needed, this structural difference might also underlie the different DNA‐binding affinity of YY2 and YY1 on their common target genes.

Hence, the mechanism of YY2 regulation on its target genes could be either competitive with YY1 or through its specific DNA‐binding site. Furthermore, the effect of YY2 regulation on the common target genes could be similar or opposite to YY1. While present studies have clearly indicated the importance of these sophisticated regulatory mechanisms, there are still crucial questions that remain to be solved, including why YY1 and YY2 could exert different functions on the same target gene through same DNA‐binding site, and which evolutionary mechanism that results in the YY2‐specific DNA‐binding site. Moreover, the specific regulatory mechanism and/or condition that regulates the balance of YY1 and YY2 regulation on their common target genes also needs further investigation.

### 1.2 The roles of YY2 in developmental biology

Even though only a few of its biological functions have been discovered, it is clear that YY2 plays crucial functions in maintaining the ESCs stemness and embryonic development. Herein, we describe the current understandings of the roles of YY2 in developmental biology.

#### 1.2.1 YY2 is crucial for stem cells maintenance and differentiation

Stemness maintenance and differentiation are two key events controlled very stringently for proper development of an organism. YY2 has been reported as a crucial factor in embryonic development, as a delicate change on its expression levels could determine cell fate. Previous report showed that CRISPR/Cas9‐based *YY2* knockout blastocyst failed to maintain its internal cell mass morphology.^9^ Knocking down of *YY2* also resulted in the depletion of mESCs culture, indicating that a basic level of YY2 is necessary for cell survival during embryonic growth.^9^ However, *YY2* overexpression suppresses the expression of various pluripotent factors in mESCs, including Oct4, Esrrb, Tet1 and Tet2, subsequently leads to the loss of mESCs stemness and pluripotency. Concomitantly, YY2 expression in mESCs is limited to a very low level to maintain its self‐renewal and pluripotency.^9^ Moreover, in mouse trophoblast stem (TS) and embryonic stem (ES) cell, YY2 could bind to some endogenous retroviral elements (ERVs) such as *murine endogenous retrovirus‐like* (*muERV‐L*) and *intracisternal A particle* (*IAP*), and increase their expression.^25^, ^27^ As ERVs could affect cellular gene expression and promote cell differentiation and morphogenesis,^28^ YY2 regulation on ERVs also suggests the importance of YY2 in promoting normal embryonic growth and organism development.

#### 1.2.2 YY2 regulates cardiovascular system development

Besides in maintaining stemness of ESCs, YY2 also has critical roles in the development and differentiation of cardiovascular system.^9^ Klar *et al* reported that YY2 is expressed with a stable rate throughout the developmental stages of the heart and lung in mouse embryos,^19^ showing the possible important role of YY2 in the development of the heart and lung. Indeed, while the roles of YY2 in heart and lung development have not been clearly revealed, a more recent study indicated that YY2 expression is important in directing mESCs differentiation to cardiovascular lineage, as demonstrated by the elevated expression of cardiovascular markers including *alpha‐myosin heavy chain* (*αMHC*), *NK2 homeobox 5* (*Nkx2*.*5*)*, myosin light chain 2a* (*MLC2a*) and *myosin light chain 2v* (*MLC2v*) in mouse embryoid bodies overexpressing *YY2*.^9^ *α*MHC is important for the development of cardiac muscle on the apex of the growing ventricle,^29^ while Nkx2.5 and MLC2v are critical for the differentiation of different regions of the heart from the early to late stages.^30^ Furthermore, MLC2a is the main constituents of myofibril in the embryonic atria which is critical for its contractional regulation.^31^ Interestingly, YY1 could also positively regulate *Nkx2*.*5* expression in cardiac progenitor cells dependent on its interaction with Gata4, a transcription factor important for heart development,^32^ and thus promotes cardiac development.^33^, ^34^ These facts suggest that YY2 and YY1 might have overlapping functions in the development of the heart. In the case of vascular restenosis development, YY2 is also involved in regulating vascular smooth muscle cells (VSMCs) proliferation and neointimal hyperplasia.^35^ Furthermore, in preventing the formation of neointimal hyperplasia due to injury, PTBP1 suppresses *YY2* expression, leading to the down‐regulation of cell cycle regulators p53 and p21, and subsequently, increases cell proliferation.^35^

YY2 also plays significant role in the proper regulation of cardiac development and homeostasis. Recently Wu *et al* showed that YY2 suppresses the expression of *MYPN*,^23^ which expression is needed for normal development of cardiac muscle and its abnormality is associated with cardiomyopathy.^36^ On the other hand, Zhang *et al* reported that elevated levels of YY2 lead to partial embryonic death, while the surviving embryos have impaired proliferation of cardiomyocytes and develop cardiomyopathy.^20^ These evidences suggest the importance of stringent YY2 regulation in the differentiation and development of the cardiovascular system. Together, YY2 plays a prominent role in regulating the development of the cardiovascular system by both directing cardiac development through elevating cardiovascular‐specific markers crucial for cardiovascular development, such as *α*MHC, Nkx2.5 and MLC2v, and at the same time, by preventing the overgrowth of cells comprising the organs through its anti‐proliferative function. This dual nature of YY2 is interesting and needs to be investigated further.

#### 1.2.3 YY2 is crucial for brain and nervous system development

Unlike the stable expression of YY2 throughout the developmental stage of the heart, YY2 expression levels are dynamically regulated in different parts of the brain during its development. Drews *et al* compared the expression levels of YY2 in each stage of development in hippocampus, neocortex and cerebellum, and revealed that YY2 expression level shows dynamic changes in the cerebellum and neocortex.^19^ From the period of intrauterine pregnancy, YY2 expression in the neocortex decreases and reaches its lowest level in the early neonatal period, while in the cerebellum, YY2 expression remains steady. From the early neonatal period to adulthood, YY2 expression increases with slow progression in the neocortex, while in the cerebellum, it rises significantly.^19^ The increasing pattern of YY2 from postnatal period to adulthood in both cerebellum and neocortex may reflect its function in neuronal development on these parts of the brain where particular parallel neuronal connections are present,^37^ and when neurogenesis and neuronal migration in the cortex are completed.^38^ These evidences suggest the importance of YY2 in stringent spatiotemporal regulation of brain development.

At the cellular level, YY2 expression is significantly lower in the neurons compared to astrocyte and microglia cells,^19^ and a more recent study further verified that YY2 plays an important role in neurite development.^22^ In mouse primary hippocampal neurons, *YY2* overexpression leads to a decreased number of neurites projection to soma and a decreased length of the longest neurite compared to the control group. In addition, YY2 overexpression in mouse neuroblastoma cell line N1E‐115 could significantly increase cellular mortality^22^; however, the expression level of two common apoptosis‐related genes, *Bax* and *Bcl‐2*, remains unchanged, reflecting that the cell death caused by overexpression of *YY2* might be induced by non‐apoptotic pathway.^22^ Hence, while the detailed molecular mechanism has not been totally unravelled, these findings showed that strict control of cellular YY2 level is a key factor in maintaining normal neurite outgrowth and cell viability.

Altogether, these results showed coherent evidences that YY2 plays an important role in developmental biology and how precise spatiotemporal control of YY2 is required for the embryonic differentiation, especially in the development and maturation of the cardiovascular and nervous system.

### 1.3 The roles of YY2 in tumour biology

Tumour cell is a type of mutant cell which loses its control, especially on its proliferation. It has specific characteristics, which are known as the hallmarks of cancer, such as accelerated cell cycle, high proliferation, metastasis and metabolic reprogramming.^39^ Recent studies have revealed that YY2 is aberrantly expressed in tumours and acts as a critical tumour suppressor gene involved in tumorigenesis and progression, as it is involved in the regulations of several hallmarks of cancer, including cell proliferation, cell cycle progression and tumour metastasis.^6^, ^7^, ^23^, ^40^ Herein, we summarize the current perspectives of the roles of YY2 in tumour biology.

#### 1.3.1 YY2 suppresses tumour cells proliferation

It is well‐known that *YY1* is an oncogene that is highly expressed in different types of cancer including lung carcinoma,^41^ liver cancer,^42^ colorectal cancer,^43^, ^44^, ^45^ melanoma,^46^ gastric cancer^47^ and prostate cancer,^48^, ^49^ and is involved in the regulation of several hallmarks of cancer.^50^ In contrast, aberrantly low YY2 expression at both mRNA and protein levels could be found in breast carcinoma, colon carcinoma and hepatocellular carcinoma.^6^, ^7^ Furthermore, based on analysis of the Human Protein Atlas database, Kaufhold *et al* revealed that down‐regulation of YY2 could also be found in prostate cancer, ovarian cancer, endometrial cancer, glioma, urothelial cancer, renal cancer, stomach cancer, cervical cancer and liver cancer.^51^ In line with its low expression level, previous studies have shown that YY2 exerts an anti‐proliferative effect, as *YY2* silencing promotes tumour cell proliferation, while *YY2* overexpression suppresses it.^23^, ^40^ Furthermore, we also showed that YY2 could affect tumour cell proliferation by regulating cell cycle progression through positive regulation on *p53* transcription.^7^ As a transcription factor that positively regulates p21 expression, p53 accumulation leads to increased p21 expression. Consequently, *YY2* down‐regulation promotes cell cycle progression and enhances tumour cells proliferation.

In addition to the aberrant regulation of YY2 expression in tumours, YY2 has also been shown to exert its tumour‐suppressive role through its regulation by post‐translational modification. A recent study demonstrated that YY2 could be methylated by histone lysine methyltransferase (SET7/9) at its lysine 247 (K247), and demethylated by lysine‐specific demethylase 1 (LSD1).^23^ In this study, Wu X. et al showed that these modifications on K247 are important for YY2 in exerting its transcriptional activity, as K247 methylation increases YY2 DNA‐binding affinity on its target genes, while K247 demethylation decreases it (Figure 3C).^23^ YY2 mutations in lysine 247 to arginine (K247R) lead to reduced YY2‐methylation by SET7/9. This subsequently attenuated the level of YY2 binding on its target genes, including *p53* and *checkpoint DNA exonuclease 1* (*RAD1*), which are crucial for preventing skin tumour development by regulating cell cycle checkpoints and DNA repair.^52^ The reduced YY2 DNA‐binding affinity also leads to the increased transcription of several oncogenes on which it acts as a negative regulator, such as *oxidized low‐density lipoprotein receptor 1* (*OLR1*), *phosphodiesterase 2A* (*PDE2A*), and *prostaglandin‐endoperoxide synthase 2* (*PTGS2*). OLR1 could activate the OLR1/c‐Myc/HMGA2 axis and promote the metastatic potential of pancreatic cancer cells,^53^ while PDE2A could promote the growth and invasiveness of malignant melanoma cells via cAMP‐PDE signalling pathway,^54^ and PTGS2 could activate the PI3K/AKT/NF‐kB signalling pathway, leading to the increase of the osteosarcoma cell migration potential.^55^ Furthermore, two types of YY2 somatic mutations K244Q and S246F (lysine 244 to glutamine and serine 246 to phenylalanine) identified in colon and skin cancer, respectively, could also decrease the methylation of YY2 at K247 and subsequently attenuated the YY2 DNA‐binding affinity to the promoter of its target genes.^23^ Cumulatively, these results emphasize the importance of YY2 post‐translational modification to the tumour‐suppressive role of YY2 by weakening its regulation on the oncogenes it suppresses, and on the tumour suppressor genes it activates. This subsequently attenuates YY2 inhibitory effect on cell proliferation and tumour growth.^23^

#### 1.3.2 YY2 suppresses tumour metastasis

Metastasis is another tumour characteristic accompanying cancer progression towards malignancy which subsequently cause systematic damage to patients. Indeed, metastasis is the culprit behind most cancer‐related deaths.^56^ Recent researches revealed that YY2 is also critical in suppressing tumour metastasis through its regulation on *AES*, a colorectal cancer (CRC) metastatic suppressor.^57^ A previous study showed that the expression of YY2 and AES in liver metastases is significantly lower than in CRC primary tumour, as YY2 enhances *AES* transcriptional activity by directly binds to its promoter.^6^ YY2 competes with YY1 in binding to the *AES* promoter, and as YY1 is abundantly expressed in tumour cells, it is enriched in the *AES* promoter region and could disturb YY2‐mediated *AES* transcription. Kakizaki *et al* demonstrated that YY2 activation effect on the *AES* promoter decreases with the increasing dose of YY1, confirming their competitive regulation in regulating *AES* promoter.^6^ This competitive inhibition leads to reduced AES level and increased metastatic activity.^6^ These results confirmed the important role of the YY2/AES axis on tumour metastasis, yet the regulatory mechanism upstream of YY2 needs to be further elucidated.

Besides *AES*, YY2 could also regulate the expression of *Cdkl2*, a factor that is involved in inducing epithelial‐to‐mesenchymal transition (EMT).^58^ EMT is a process in which tumour cells lose their epithelial characteristics and gain mesenchymal characteristics, and is the first step in the metastasis cascade. These facts clearly point to the possibility that YY2 could suppress not only tumour cell proliferation, but also tumour metastasis.

#### 1.3.3 The roles of YY2 in tumour cells metabolic reprogramming

Metabolism is a fundamental biological process that supports every living cell, including tumour cells. To support their highly proliferative characteristic, tumour cells alter their metabolic pathway to fulfil the demand in energy and building blocks of macromolecules composing the cells.^39^ Tumour cells enhance their glucose uptake and glycolytic rate, shifting the glucose metabolic pathway from glycolysis followed by oxidative phosphorylation to glycolysis followed by fermentation even in the condition of sufficient oxygen supply.^59^, ^60^ They also promote their lipid accumulation, as lipid is not only a component of cellular and organelles membrane, but also crucial for energy metabolism and signal transduction for various biological functions.^61^, ^62^ Furthermore, they enhance the rate of pentose phosphate pathway (PPP), which provides them with nucleotides precursors and NADPH, a cellular reductant critical for suppressing increased cellular ROS due to rapid proliferation, and for enhancing lipid biosynthesis.^63^, ^64^ Recently, we found that YY1 could alter tumour cells metabolic reprogramming by regulating the transcription of key rate‐limiting enzymes, including *GLUT3* and *G6PD*, which are crucial in glucose uptake and stimulation of the pentose phosphate pathway, as well as by regulating *PGC‐1β*, which is crucial for tumour cells lipid metabolism.^42^, ^43^, ^44^ While the specific roles of YY2 need to be examined more extensively, some studies have hinted that YY2 might affect tumour metabolism. By using gene ontology analysis, Wu *et al* reported that YY2 negatively regulates genes related to cellular cholesterol and sterol metabolism in HeLa cells.^23^ In addition, Chen *et al* found that YY2 negatively regulates a wide variety of genes in the tumour cell, including those involved in lipid metabolism. They found that YY2 inhibits the expression of *cluster of differentiation 36* (*CD36*, also known as *SR‐B2*), while YY1 enhances it.^40^ CD36 is a fatty acid translocase which promotes the absorption of long‐chain fatty acids (LCFA) and activates PPARγ; thereby promotes lipid accumulation in hepatocarcinoma cells.^65^, ^66^, ^67^, ^68^ Thus, these evidences suggest the possibility that YY2 negatively regulates tumour cells lipid accumulation.

Furthermore, as described above, YY2 could enhance *p53* transcription,^3^, ^69^ and p53 is crucial for tumour metabolic reprogramming.^70^ p53 has been known to regulate tumour cells glucose metabolism by suppressing the expression of *GLUT3*, as well as *TP53‐induced glycolysis and apoptosis regulator* (*TIGAR*) and *hexokinase 2* (*HK2*), which plays important roles in glycolysis.^71^, ^72^ Furthermore, TIGAR could also protect tumour cells against oxidative stress, while HK2 is crucial for energy production, preservation of mitochondrial integrity and cell survival.^73^ Together, these suggest that YY2 might also be involved in regulating glucose metabolism in tumour cells.

Overall, while the understandings regarding its molecular mechanisms have not been totally elucidated yet, the findings regarding its role in regulating tumour cells proliferation, metastasis and metabolic reprogramming point out the importance of YY2 as a tumour‐suppressive gene. It is also noteworthy that in the regulation of some genes that promote tumour development such as *c‐Myc*, *c‐Fos*, and *CXCR4*, the effect of YY2 depends on its dose, with mechanisms remain to be unravelled. Nevertheless, the fact that YY2 is down‐regulated in tumour cells indicates the possibility of using YY2 as a marker of tumour progression and prognosis.

### 1.4 The roles of YY2 in immune biology

Immune system is an essential host defence that functions by recognizing and eradicating pathogens and other foreign molecules. The disturbances of the immune system lead to many diseases, including severe infections, tumours, allergies and autoimmune diseases.^74^ On the other hand, immune system is also an important and indispensable component of tumour microenvironment, which is crucial for promoting tumorigenesis and development.^75^ Deregulation of genes important in immune responses, such as *IL‐4* and *IFN‐β*, promotes tumour growth, invasion, metastasis and chemotherapy resistance.^76^, ^77^ Secretion of immunoregulatory cytokines such as IL‐4 leads to the induction of cathepsin protease activities in tumour‐associated macrophage, which then promotes pancreatic tumour growth.^76^ Meanwhile, increased IFN‐*β* level in triple‐negative breast cancer could promote tumour‐infiltrating lymphocytes activity and repress tumour cells’ CSC‐like properties.^77^ As described below, recent studies reported that YY2 might regulate the expression of these immunoregulatory cytokines, suggesting the possible role of YY2 in immune activity in carcinoma.^8^, ^21^

Type I interferon is a class of pleiotropic cytokines consists of IFN‐*α* and IFN‐*β* originally found to interfere with the replication of viral and bacterial.^78^, ^79^ Klar *et al* showed that both YY2 and YY1 DNA‐binding sites are found in the far‐upstream region (−2kb and −3kb) of mouse *IFN‐β* gene promoter and regulate its expression level with different regulatory effect. They found that while YY1 negatively regulates *IFN‐β* expression, YY2 co‐expression with YY1 alleviates YY1 suppression on *IFN‐β*. However, the expression level of IFN‐*β* does not significantly change when YY2 was overexpressed alone, plausibly due to that the endogenous levels of YY2 might be sufficient to support the induction of IFN‐*β*. These unique regulations of YY1 and YY2 on *IFN‐β* promoter indicate that they might competitively bind to the same DNA‐binding sites and play antagonistic role in regulating the expression of *IFN‐β*.^21^ Recent studies demonstrated that IFN‐*β* has tumour‐suppressive functions in several types of carcinomas, including triple‐negative breast cancer (TNBC), human colorectal carcinoma and oral squamous cell carcinoma, by activating cytotoxic T lymphocyte in the tumour microenvironment.^80^, ^81^, ^82^, ^83^ Activated cytotoxic T lymphocyte will then up‐regulate the host cancer immunosurveillance activity and suppress tumour development.^77^, ^84^, ^85^ These facts point to the possibility of YY2 involvement in activating immunosurveillance and performing its tumour‐suppressive effect by maintaining the level of IFN‐*β*.

IL‐4 is a pleiotropic cytokine that is important in the regulation of immune response. It plays a crucial role in regulating the differentiation of antigen‐stimulated naive T cells towards T helper 2 (Th2) cells, which leads to the generation of immune response against helminthic diseases.^86^, ^87^ Aberrant IL‐4 expression is also correlated with increased immunoglobulin E production that underlies allergic disorders and asthma.^88^, ^89^, ^90^, ^91^ Furthermore, IL‐4 could also activate M2 macrophages, which in turn activate JNK signalling pathway that leads to increased pro‐inflammatory responses.^92^ Recently, Lee et al found that YY2 suppresses *IL‐4* promoter activity, while YY1 enhances it. Co‐expression of YY1 and YY2 attenuates YY1 promotion effect on *IL‐4* promoter activity, most plausibly due to their competitive binding to *IL‐4* promoter.^8^ Thus, YY2 and YY1 might play antagonistic role in regulating *IL‐4* transcription. Given that IL‐4 enhances T cells differentiation into Th2 and promotes Th2 immune responses, YY2‐mediated regulation on IL‐4 implies its possible regulation on type 2 immune response and the inflammatory response of the phagocytes. Moreover, as IL‐4 contributes to tumour growth, metastasis and chemotherapy resistance, this fact indicates that IL‐4 might be crucial for YY2‐mediated tumour suppression.^76^, ^93^, ^94^, ^95^ However, despite that present understanding suggests its possible involvement, the detail roles and regulatory mechanism of YY2 in immune system, especially in tumour immunity, need further investigations.

## 2 CONCLUSION AND PERSPECTIVES

YY2, as a more recently discovered homolog of YY1, possesses structural similarity to YY1 and thus might have functional similarity with YY1 in regulating target genes. However, as shown in Table 2, recent studies have shown that YY2 also has unique, or even opposite functions to that of YY1. Although YY2 and YY1 share some similar DNA‐binding sites, YY2 also possesses its own specific target genes due to its specific DNA‐binding sequence. These lead to the complexity of YY1 and YY2 regulation on their target genes, suggesting possible different patterns: (1) YY2 and YY1 coordinately regulate gene expression on different degrees; (2) YY2 competes with YY1 for the common DNA‐binding site of the same target genes; (3) YY2 has its own target genes, which is due to the presence of YY2‐specific DNA‐binding site that is different from that of YY1. While the reasons and mechanisms underlying these common and specific regulations of YY family genes remain to be investigated, the structural difference between YY2 and YY1 proteins, with a more ordered characteristic of the N‐terminus and a lack of acidic domain in YY2 proteins, might be crucial. Another intriguing question that needs to be explored further is whether YY2 could also function as a post‐translational regulator, as YY1 could regulate some of its target genes, including *AKT*, *p53* and *HIF‐1α*, through post‐translational modifications.^41^, ^96^, ^97^ While the similarity of YY2 to YY1 protein confers this possibility, there is no report regarding post‐translational regulation by YY2 at present.

**TABLE 2:** Biological functions of YY2 and YY1

Functions	Genes	Regulation by YY1	Regulation by YY2	References
Oncogene	*c‐Fos*	Repression	Activation*	^3^
	*c‐Myc*	Activation	Activation (L)^***^ Repression (H)	
		
Tumour suppressor	*p53*	Repression	Activation	3, 7
	*p21*	Repression	Activation	
Tumour angiogenesis	*CXCR4*	Repression	Activation (L)^***^ Repression (H)	^3^
		
Epithelial‐Mesenchymal Transition	*Cdkl2*	Not clear^****^	Not determined^*^	^25^
	*Gcnt2*	Not clear^****^	Not determined^*^	
Stemness and pluripotency	*Esrrb*	Not clear	Repression	9, 99
	*Tet1*	Not clear^****^	Repression	
	*Tet2*	Not clear^****^	Repression	
	*Oct4*	Activation	Repression	
Differentiation and morphogenesis	*IAP*	Not clear^****^	Activation	^25^
	*muERV‐L*	Not determined^*^	Activation	
Cardiovascular	*MLC2a*	Not clear^****^	Activation	9, 23, 100
development	*MLC2v*	Not clear^****^	Activation	
	*MYPN*	Not clear^****^	Repression	
	*NKx2*.*5*	Activation	Activation	
	*αMHC*	Repression	Activation	
Cell cycle regulation	*p21*	Repression	Activation	3, 7, 40
	*CDC2*	Repression	Repression	
	*p53*	Repression	Activation	
	*c‐Myc*	Activation	Activation (L)^***^ Repression (H)	
	
Pro‐apoptosis	*BCL2L11*	Slight repression	Repression	3, 40, 69
	*p53*	Repression	Activation	
	*c‐Myc*	Activation	Activation (L)^***^ Repression (H)	
	
Tumour metastasis	*Fras1*	Not clear^****^	Not determined^*^	23, 25, 101
	*OLR1*	Not clear^****^	Repression	
	*PDE2A*	Not clear^****^	Repression	
	*PTGS2*	Activation	Repression	
Suppresses tumour metastasis	*AES*	Activation with co‐activator	Activation	^6^
		
Tumour metabolism	*CD36*	Activation	Repression	40, 69
	*p53*	Repression	Activation	
Immune responses	*IL‐4*	Activation	Repression	^8^
Immunosurveillance	*IFN‐β*	Repression	Compete with YY1	^21^
	*muERV‐L*	Not determined^*^	Activation	^25^

* The reference only showed that YY2 binds to the promoter of the indicated gene using chromatin immunoprecipitation assay.

** YY2 activity in regulating the indicated gene is more prominent at low dose.

*** YY2 activates the transcription of the indicated gene at low dose, and suppresses it at high dose. L, low dose of YY2; H, high dose of YY2.

**** There are no references indicated whether YY1 could regulate this gene at present.

A crucial problem raised in the studies regarding YY family is the cross‐reactivity between antibodies targeting YY1 and YY2 due to their sequential similarities^3^, ^98^; however, a comparative study by Kakizaki *et al* showed that some YY family antibodies do not show cross‐reactivity.^6^ Furthermore, as summarized in Table 3, most of the previous studies describing the functions and molecular mechanisms of YY2 have provided evidences from several aspects, such as mRNA, protein, cellular and phenotypes levels. Moreover, these studies used more than one experimental methods including those which do not use antibody or antibodies which are not relevant with the cross‐reactivity between YY1 and YY2,^3^, ^9^, ^40^ and most of the studies validated the specificity of YY2 antibodies they used. Nevertheless, while it is clear that YY2 has its own, unique biological functions, attention should be paid regarding the specificity of the antibody used for studying YY2, and for targeting YY family for treating related diseases.

**TABLE 3:** Specificity confirmation of YY2

References No.	Main experiments	Use antibodies/not	Specificity confirmation**	Methods used for validating antibodies specificity
^3^	EMSA, GST‐pull down, western blotting, reporter assay	Yes; anti‐GST‐YY2 antibody (made by themselves), anti‐Gal4, anti‐His, anti‐Flag antibodies	Specific	Western blotting and immunofluorescence
^21^	EMSA, Yeast one‐hybrid, reporter assay	Yes; anti‐Flag antibody	Specific	Western blotting
^1^	in situ hybridization	No	Not relevant	N/A
^2^	Gel shift assay	Yes; anti‐GST antibody	Not relevant	N/A
^19^	in situ hybridization, qRT‐PCR	No	Not relevant	N/A
^24^	qRT‐PCR, DNA demethylation	No	Not relevant	N/A
^40^	qRT‐PCR, western blotting, EMSA, immunoprecipitation assay, Gene Set Enrichment Analysis	Yes; anti‐YY2 antibodies (Santa Cruz, sc‐47637 and sc‐47635)	Specific	Western blotting
^27^	ChIP assays, qRT‐PCR	Yes; anti‐YY2 antibody (made by themselves), anti‐HA antibody,	Specific	Immunoprecipitation assay
^22^	qRT‐PCR, reporter assay, DNA demethylation, western blotting	Yes; anti‐YY2 antibody (Santa Cruz, sc‐377008)	Specific	Immunoprecipitation assay
^6^	Reporter assay, western blotting, ChIP, EMSA, immunohistochemistry, immunofluorescence, and immunoprecipitation assays	Yes; anti‐YY2 antibodies (Santa Cruz, sc‐374455, ab116507, sc135197)	Specific	Immunoprecipitation assay
^8^	Reporter assay, DNA affinity precipitation assay, western blotting	Yes; anti‐YY2 antibodies (sc‐135197) anti‐Myc antibody	Specific	Western blotting
^25^	ChIP, qRT‐PCR	Yes; anti‐HA antibody anti‐YY2 antibody (made by themselves)	Specific	Immunoprecipitation assay
^9^	Western blotting, ChIP‐sequencing	Yes; anti‐YY2 antibody (Santa Cruz, sc‐377008)	Specific	Western blotting
^69^	Western blotting, ChIP, immunohistochemistry, immunoprecipitation	Yes; anti‐YY2 antibody (Abcam, Ab116507)	Specific	Western blotting
^23^	EMSA, western blotting, ChIP‐sequencing	Yes; anti‐YY2K247me1 and anti‐YY2K247pan antibodies (GenScript), anti‐Flag, anti‐HA antibodies	Specific	Western blotting
^10^	Structural analysis of YY2	No	Not relevant	N/A
^35^	Western blotting	Yes; YY2 antibody (Santa Cruz, product number N/A)	Specific	Western blotting
^20^	Western blotting	Yes; YY2 antibody (Santa Cruz, sc‐374455), anti‐HA antibody	Specific	Validated in Ref. No.6

* Evidence of the specificity of YY2 antibody as shown in each paper; N/A, not available; Not relevant, antibody used is not relevant with YY1/YY2 cross‐reactivity.

**FIGURE 4:**
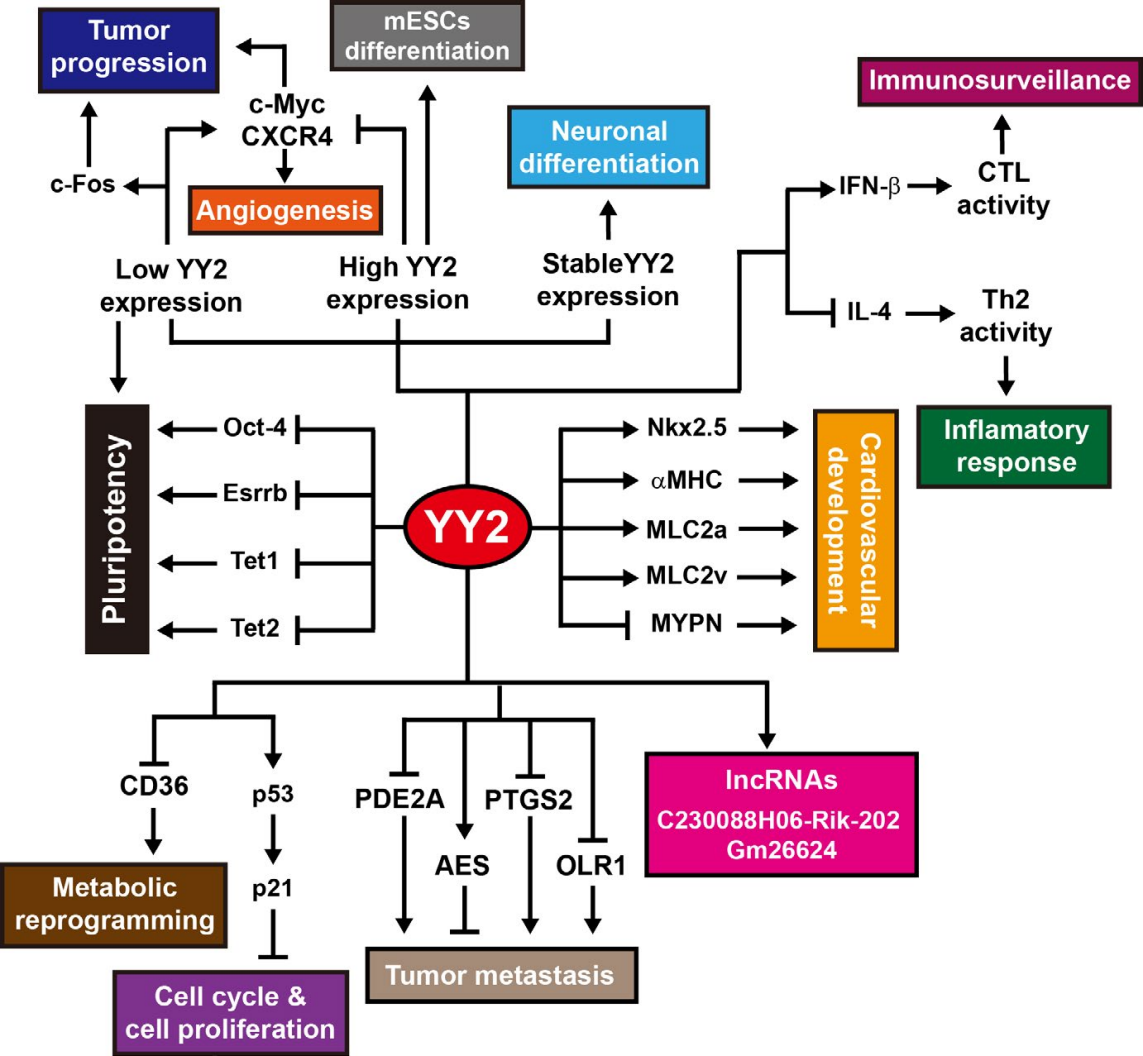
Summary of physiopathological functions of YY2. YY2 is involved in several physiopathological events. Low level of YY2 is crucial for maintaining the pluripotency of embryonic stem cells, and overexpression of YY2 drives cells toward differentiation. YY2 is also involved in the development of organs such as heart and neurons, as well as immune response. Furthermore, it acts as a tumour suppressor gene by suppressing tumour cells proliferation, tumour metabolism, and tumour metastasis

Despite the limited reports of its biological and pathological functions, some studies have shown that YY2 is crucial in embryonic development, tumorigenesis and immune response (Figure 4). However, there are unravelled questions that need further investigation, especially the mechanism of how YY2 is down‐regulated in primary tumours by its upstream regulators. Furthermore, while the basal level of YY2 is important for maintaining ESCs stemness, elevated YY2 expression results in the loss of stemness, and excess YY2 expression impairs cell proliferation and induces neurological and cardiac development disorders. These imply the importance of the dual nature of YY2, which requires a precise spatiotemporal as well as expression level regulations during embryonic development. Overall, a more comprehensive studies including omics analysis as well as *YY2* transgenic and/or knockout animals are absolutely necessary for completely unravelling its detailed phenotypic and molecular mechanism.

In conclusion, the broad range of YY2 functions in various physiological processes could be the base for future studies aimed to define and elucidate further the unique characteristics of YY2, as well as its potential as prognosis markers and therapeutic targets for diseases.

## CONFLICT OF INTEREST

The authors declare that there is no conflict of interest.

## AUTHOR CONTRIBUTION

Lang Li: Formal analysis (equal); Investigation (equal); Visualization (equal); Writing‐review & editing (equal). Yanjun Li: Formal analysis (equal); Investigation (equal); Visualization (equal); Writing‐review & editing (equal). Ian Timothy Sembiring Meliala: Formal analysis (equal); Investigation (equal); Visualization (equal); Writing‐review & editing (equal). Vivi Kasim: Funding acquisition (equal); Investigation (equal); Supervision (equal); Resources (equal); Writing‐original draft (lead); Writing‐review & editing (equal). Shourong Wu: Funding acquisition (equal); Investigation (equal); Supervision (equal); Writing‐review & editing (equal).
